# Two decades of warming increases diversity of a potentially lignolytic bacterial community

**DOI:** 10.3389/fmicb.2015.00480

**Published:** 2015-05-20

**Authors:** Grace Pold, Jerry M. Melillo, Kristen M. DeAngelis

**Affiliations:** ^1^Microbiology Department, University of MassachusettsAmherst, MA, USA; ^2^Graduate Program in Organismic and Evolutionary Biology, University of MassachusettsAmherst, MA, USA; ^3^Marine Biological LaboratoriesWoods Hole, MA, USA

**Keywords:** chemically complex carbon, climate change, microbial ecology, soil organic matter, Bio-Sep beads, lignin degradation, *in-situ* enrichment

## Abstract

As Earth's climate warms, the massive stores of carbon found in soil are predicted to become depleted, and leave behind a smaller carbon pool that is less accessible to microbes. At a long-term forest soil-warming experiment in central Massachusetts, soil respiration and bacterial diversity have increased, while fungal biomass and microbially-accessible soil carbon have decreased. Here, we evaluate how warming has affected the microbial community's capability to degrade chemically-complex soil carbon using lignin-amended BioSep beads. We profiled the bacterial and fungal communities using PCR-based methods and completed extracellular enzyme assays as a proxy for potential community function. We found that lignin-amended beads selected for a distinct community containing bacterial taxa closely related to known lignin degraders, as well as members of many genera not previously noted as capable of degrading lignin. Warming tended to drive bacterial community structure more strongly in the lignin beads, while the effect on the fungal community was limited to unamended beads. Of those bacterial operational taxonomic units (OTUs) enriched by the warming treatment, many were enriched uniquely on lignin-amended beads. These taxa may be contributing to enhanced soil respiration under warming despite reduced readily available C availability. In aggregate, these results suggest that there is genetic potential for chemically complex soil carbon degradation that may lead to extended elevated soil respiration with long-term warming.

## Introduction

The size of the soil carbon pool exceeds that of atmospheric and terrestrial vegetation carbon pools combined (Jobbágy and Jackson, 2000), making the fate of soil carbon a key variable in global climate models (McGuire et al., 2001; Wieder et al., 2013). Environmental perturbations that reduce the soil carbon pool or affect the microbes which process it may exacerbate soil carbon loss if this reduction in soil carbon stock feeds back to the initial stressor, as may occur under climate change (Davidson and Janssens, 2006). Indeed, experimental warming almost ubiquitously increases soil respiration (Rustad et al., 2001; Lu et al., 2013). Such increased decomposition is likely to be the consequence of some combination of both the direct effects of temperature on the organisms, and the indirect effects of altered community and/or soil organic matter composition (Bradford et al., 2008, 2010).

Accelerated microbial enzyme kinetics in response to warming can lead to increased soil respiration. In order to access polymeric carbon outside the cell, soil microorganisms produce extracellular enzymes that are affiliated with cell membrane surfaces or extracellular polysaccharides, or that are released into the environment (Wallenstein et al., 2011). These extracellular enzymes may have thermal optima greater than the temperatures they experience in the soil (Parham and Deng, 2000; Yan et al., 2010; Schipper et al., 2014), such that moderately elevated temperatures favor increased activity, as predicted by the Arrhenius equation (Stone et al., 2012; Baldrian et al., 2013). Physical explanations proposed for this phenomenon include increased flexibility of the active site and increased desorption of enzyme and substrate from mineral surfaces (Conant et al., 2011; Wallenstein et al., 2011). From a biological perspective, increased metabolic rates may enable greater enzyme production, since intracellular metabolism of soil microbiota is also responsive to temperature (Wallenstein et al., 2011). In addition, substrates that were previously degraded at relatively low rates due to the high activation energy of the decomposition reaction may now be more readily degraded, although it is unclear if this holds for soil enzymes (Davidson and Janssens, 2006; Baldrian et al., 2013; Erhagen et al., 2015).

In contrast to these direct kinetic effects of warming on microbial activity, this research examines how changes in microbial physiology or community structure may relate to elevated rates of soil carbon loss under warming. Warming may make microbial growth less efficient, likely through some combination of facilitating microbial access to lower-quality substrate pools (Devêvre and Horwáth, 2000) that require greater investment of resources to process, and by causing physiological changes in the organisms (Manzoni et al., 2012). Since only some soil bacteria have the capacity to rapidly respond to the presence of the most chemically complex compounds (Goldfarb et al., 2011), the reduction in the quality of soil carbon pools seen in some warming studies (Melillo et al., 2002; Bradford et al., 2008; Xu et al., 2012) may affect community structure. Indeed, although degradation of polymeric litter carbon is generally considered a fungal process (Boer et al., 2005; Moore-Kucera and Dick, 2008; Schneider et al., 2010, 2012; Baldrian et al., 2012), fungal:bacterial ratios have declined in a number of warming studies (Frey et al., 2008; Flury and Gessner, 2011; Zelikova et al., 2012; Sistla et al., 2013). Given the wide functional diversity and broad array of terminal electron acceptors they can use, bacteria are key decomposers of complex carbon under certain conditions (DeAngelis et al., 2011), but the extent to which these bacterial abilities are important for accelerated carbon cycling under extended warming is still unclear.

At our research site in a temperate deciduous forest in New England, 23 years of artificially warming soils has led to considerable changes in the stocks and flows of soil carbon. After 15 years, soil organic matter and carbon available to microbes had declined (Bradford et al., 2008; Frey et al., 2008). Soil respiration at this site has shown an initial increase in respiration with warming, accompanied by a decrease in fungal biomass (Frey et al., 2008), which disappeared after a decade (Melillo et al., 2002). Recently, we also observed substantial changes in microbial communities with warming, including a more than 80% increase in Alphaproteobacteria ribosomal RNA gene counts (DeAngelis et al., 2015). Using ^13^C-phenol and glucose, Frey and colleagues found evidence that warming has selected for a community specifically adapted to the more efficient utilization of structurally stable carbon at elevated temperatures (Frey et al., 2013). Research at an adjacent warming study indicates that while microbes are adapted to more rapid growth at higher temperatures, they are still limited overall by access to readily available carbon (Rousk et al., 2012). Together, these results indicate that increased utilization of chemically complex carbon may be driving the observed changes in carbon cycling at this site.

Here we evaluate whether the structure and enzymatic potential of the microbial community associated specifically with chemically complex carbon decomposition at this site has changed, using the heteropolymeric compound Kraft lignin as a proxy for chemically complex carbon. Lignin, which comprises 10–30% of leaf litter biomass (Aber et al., 1990), is degraded primarily by non-specific oxidative enzymes which may also break down soil organic matter (Fontaine et al., 2003; Creamer et al., 2015). By baiting soil microbes with lignin, we tested the hypothesis that experimental warming treatment has increased the diversity of the lignin-associated bacterial community.

## Materials and methods

### Experimental design

Our experiment was conducted within a long-term warming experiment in a mixed hardwood stand at the Harvard Forest LTER, Petersham, MA. At this site, soil temperatures have been artificially raised by 5°C using buried resistance cables since 1991 (Melillo et al., 2002). The dominant trees are *Acer rubrum*, *Betula papyrifera*, *Quercus velutina*, and *Acer pensylvanicum*, and the soils are coarse-loamy inceptisols (Peterjohn et al., 1994). The climate is temperate moist, with mean monthly temperatures ranging from −6°C in January to +20°C in July and a mean annual precipitation of 118 cm since the onset of the experiment (Boose and Gould, 1999; Boose, ongoing).

To each of four replicate heated and four replicate disturbance control plots, we horizontally deployed separate pouches containing lignin-amended (Sigma no. 471003) or unamended Bio-Sep bead (Microbial Insights, Knoxville TN) pouches (details in next section). Pouches were deployed as either “surface” bags, or as “subsurface” bags. Surface bags were placed on the surface of the soil under the leaf litter layer. Subsurface bead bags were buried in the soil between the organic horizon and the mineral soil. Where the depth of the organic horizon exceeded 3 cm, the beads were deployed to a depth of 3 cm. Bags were positioned in pairs, such that the lignin amended and unamended bead bags were side-by-side, but the surface and subsurface bead bags were at an approximate horizontal distance of at least 5 cm. We incubated a total of 32 bead pouches at our research site. Bags were deployed on August 5, 2013 and remained buried until October 23, 2013, a total of 11 weeks. This coincides with the seasonal period over which the greatest difference in soil respiration between heated and control plots can be seen (Melillo et al., 1999), and when the largest influx of complex plant litter to the soil occurs (Bowden et al., 2014).

### Bio-Sep bead pouches

Bio-Sep beads are ~3–4 mm diameter porous spheres consisting of activated charcoal (25%) in a Nomex matrix (75%) (Williams et al., 2013). The beads are biochemically inert but are able to sorb nutrients. Selected substrates can be attached to the surfaces of the beads by covalent bonding via a proprietary method that enables microbial access to substrates while preventing leaching. Bio-Sep beads have been used in aquatic systems (Anderson et al., 2003; Peacock et al., 2004; Sublette et al., 2006; Baldwin et al., 2008; Williams et al., 2013) as well as terrestrial systems (DeAngelis et al., 2011; Omotayo et al., 2011) to monitor microbial activity. Bead pouches used in this study consisted of 5 g (~20 ml) of Kraft lignin amended or unamended beads in an 8 cm diameter circular window screen mesh pouch (Phifer silver gray fiberglass screen, product 4788811608, approximately 1 mm mesh size) that was heat-sealed. These pouches were then encased in a 9.5 × 9.5 cm square hardware cloth (YardGuard® 1/4 inch mesh (23 gage) with galvanized zinc coating, product 308231B), which kept the beads approximately two layers thick.

### Enzyme assays

Total oxidative enzyme assays were completed using 25 mM L-DOPA +0.3% hydrogen peroxide (Saiya-Cork et al., 2002). Slurry was prepared by vortexing 2 g of beads in 40 ml pH 4.7 Modified Universal Buffer (Östling and Virtama, 1946). An equal volume of substrate was added to each well. Plates were incubated at room temperature (23°C) in the dark for 48 h, and absorbance of a 50 μl aliquot was measured at 460 nm at five points during this time. Activity was calculated as the maximum change in absorbance over any three time points and standardized to total cell count assuming an extinction coefficient of 7.9 (Bach et al., 2013). Direct cell counts were completed on the initial bead slurry after staining with DAPI (4′,6-diamidino-2-phenylindole) (15–20 fields per sample); enzyme assays are reported as rates of cell normalized substrate converted per hour, and per gram of field-moist beads.

### DNA extraction

DNA was extracted between three and seven times from 0.3 g Bio-Sep beads (depending on yields) following a modified CTAB bead-beating procedure in tubes with three 5 mm glass beads (DeAngelis et al., 2010). To clear residual phenol, all extractions for a given sample were pooled and brought up to 200 μl with sterile Tris-Cl (pH 8.5), washed with 25:1 chloroform:isoamyl alcohol, and precipitated in 100% cold ethanol and sodium acetate (pH 5.2) at a final concentration of 0.3 M. Samples were desalted with 70% ethanol. DNA quality was verified using a NanoDrop 2000C (Thermo Scientific, Inc., Waltham MA), and quantified using the Quant-iT™ PicoGreen® dsDNA Assay Kit (Invitrogen).

### 16S rRNA gene sequencing

DNA was prepared for sequencing using a previously published method with a few modifications (Caporaso et al., 2011). Briefly, the V4 region of the 16S rRNA gene in the DNA template was amplified in triplicate using the primers 515F (5′-GTGCCAGCMGCCGCGGTAA-3′, Turner et al., 1999) and 806R (5′-GGACTACHVGGGTWTCTAAT-3′, Caporaso et al., 2011) with sequencer adapters and sample-specific Golay barcodes on the forward primer. The 25 μl reaction mix contained 2.5 μl of each primer at a final concentration of 200 pM, 1.875 units of Takara ExTaq polymerase, 25 μg BSA, 200 μM each dNTPs, and 10 ng template. The amplification cycle consisted of an initial denaturation at 95°C for 5 min followed by 31 cycles of 30 s at 95°C, 25 s at 50°C, 120 s at 72°C and a final elongation of 10 min at 72°C. After verifying successful PCR amplification using agarose gel electrophoresis, technical triplicate reactions were pooled for cleanup using Qiagen MinElute kit. These were then quantified using PicoGreen® and assessed for quality using Nanodrop. Equimolar quantities of each of the samples were pooled into a single tube for paired-end 2 × 150 bp Illumina MiSeq sequencing at the Molecular Biology Core Facility at the Dana-Farber Cancer Institute.

FastQC was used to check for overall sequencing quality (Andrews, 2010). Paired end reads were subsequently merged using FLASH with default parameters except sequence length, which was limited to 253 ± 1 bp (Magoč and Salzberg, 2011). All subsequent stages of the sequence data processing were completed in QIIME v. 1.8.0 (Caporaso et al., 2010b). Demultiplexing and initial quality filtering were completed with a minimum Phred score of 20. We picked operational taxonomic units (OTUs) using QIIME's subsampled open reference picking protocol using UClust (Edgar, 2010) to bin sequences into OTUs at 99% identity using RDP (Wang et al., 2007) with Greengenes (V13.5, May 2013, McDonald et al., 2012; Werner et al., 2012) as the reference database. Sequences were aligned against Greengenes using PyNAST (Caporaso et al., 2010a) and an amplicon region specific lanemask. Chimeric sequences were identified using ChimeraSlayer (Haas et al., 2011). After removing doubletons from the dataset, we rarefied the community (Magurran and McGill, 2011; Unterseher et al., 2011). This process reduced the 8.5 million reads and 64,351 OTUs to 2.4 million reads and 56,835 OTUS in 32 bacterial (56,815 OTUs, 99.998% of reads) and 2 archaeal phyla (20 OTUs) (Table S1). All sequences are available under BioProject ID PRJNA242968.

### TRFLP of fungal its region

Terminal restriction fragment length polymorphism analysis was used to assess whether 23 years of warming has affected lignin-amended and unamended bead-associated fungal communities. The fungal-specific (Klamer et al., 2002) forward and reverse primers (ITS1F; 5′-CTTGGTCATTTAGAGGAAGTAA-3 /ITS4; 5′- TCCTCCGCTTATTGATATGC-3′, Gardes and Bruns, 1993) were both labeled with a fluorescent dye (ITS1F-FAM and ITS4-VIC, respectively). The 25 μl reaction mix contained 2.5 μl of each primer at a final concentration of 300 pM, 1.875 units of Takara ExTaq polymerase, 25 μg BSA, 200 μM each dNTPs, and 10 ng template DNA. Reaction conditions were: 1 min at 95°C, followed by 35 cycles of 95°C for 1 min, 51°C for 1 min, and 72°C for 3 min, with a final extension at 72°C for 8 min. PCR product was digested with 20U HaeIII restriction enzyme (Thermo Scientific). Approximately 70 ng digested PCR product was mixed with 0.5 μl of the GeneScan 1200 LIZ size standard (ABI) and 7.7 μl deionized formamide, and submitted for sequencing on an ABI 3130XL. TRFLP profiles were checked for quality and peaks heights >50 RFU were extracted using the Local Southern method in Peak Scanner Software v. 2.0 (Life Technologies) (Blackwood et al., 2003). Peak sizes were then rounded to the nearest integer and forward and reverse fragments separated to normalize peak heights by the sum of peak heights for a dye in a sample, then the two data sets were combined for analysis. This resulted in an average of 64.7 peaks per sample, with a total of 458 different TRFs across samples.

### Identification of potentially lignolytic bacteria

In order to identify bacterial genera capable of lignin degradation, we used a PubMed literature search with the keywords “lignin degradation” +bacteria on 13th March 2014, and then manually filtered the results to exclude irrelevant content (i.e., papers on fungi, or on other biopolymer degradation) and to include references cited within papers. We also included other papers that were not included in this search but were already familiar to us. The genera appearing in this search but not found in our dataset were *Aeromonas* (Gupta et al., 2001), *Aneurinibacillus* (Chandra et al., 2007), *Azotobacter* (Morii et al., 1995), *Cladosporium* (Ji et al., 2014), *Desulfovibrio* (Kim et al., 2009), *Enterobacter* (DeAngelis et al., 2013), *Kocuria* (Parshetti et al., 2011), *Microbulbifer, Sagittula* (González et al., 1996; Chen et al., 1997) *Micrococcus* (Taylor et al., 2012), *Pantoea* (Xiong et al., 2014), *Thaurea* (Kim et al., 2009), *Thermobifida* (Chen et al., 2013), and *Xanthomonas* (Odier and Monties, 1978).

### Statistical analysis

The experimental design included four biological replicates unless otherwise noted, with 16 technical replicates for enzyme assays. All statistical analyses were completed in R (R Development Core Team, 2008). Data were tested for normality and homogeneity of variances using the Shapiro-Wilk test (Royston, 1982) and Brown–Forsythe tests (Fox, 2008), respectively, with a *p*-value cutoff of 0.1. For data which did not meet parametric assumptions, the function boxcoxfit (geoR, Ribeiro and Diggle, 2001) was used to determine the optimal lambda for power transformation. Lignin-amended and unamended bead pouches for each plot and soil depth were subsequently treated as paired samples for analysis in three-way full factorial ANOVA with warming treatment, soil, and lignin amendment as main effects. Significant interaction effects were further assessed with Tukey's Honestly Significant Difference test (Tukey, 1949).

To determine whether lignin beads recruited known lignolytic organisms, relative abundance (percent reads) of genera with proposed lignin-degraders were pooled by soil effect and warming treatment. OTUs without genus assignment were removed before analysis, and samples were normalized by sum to account for different degrees of genus-level assignment before calculating *p*-values with a paired Wilcoxon rank-sum test. Reported *p*-values are corrected for multiple testing using Benjamini-Hochberg correction (Benjamini and Hochberg, 1995).

Parameters of alpha diversity were calculated using the vegan package (Oksanen et al., 2013) with criteria outlined in Magurran and McGill as a guide for selecting uncorrelated metrics (Magurran and McGill, 2011). Since some diversity metrics are non-linear (e.g., the Shannon-Weiner index), we converted these values to effective number of species using N_eff_ = exp(H) prior to comparison (Jost, 2006). Faith's phylogenetic diversity (Faith, 1992) was calculated using picante (Kembel et al., 2010).

TRF profiles of the fungal community were compared using Hellinger distance (Legendre and Gallagher, 2001), and variables driving community structure were assessed using a permutational manova (Anderson, 2001). For the bacterial community, we completed principal coordinates analyses on weighted UniFrac distances (Lozupone and Knight, 2005) of the prokaryotic community using the phyloseq package (McMurdie and Holmes, 2013). Experimental factors driving relative abundance of major phyla and classes were assessed using step-down regression with Aikake's Information Criterion to select the best model while minimizing parameters (stepAIC, MASS, Venables and Ripley, 2002).

Dominant OTUs responsible for driving observed community trends in lignin-amendment and heating treatment were identified using paired *t*-tests or an indicator species test (Cáceres and Legendre, 2009; Roberts, 2013) with the Benjamini-Hochberg correction for multiple comparisons (Benjamini and Hochberg, 1995). Only OTUs shared by both lignin and unamended beads were considered in this analysis so as to separate ability to grown in the different bead types from a warming effect. Trees were drawn in iTOL (Letunic and Bork, 2007).

## Results

### Long-term warming recruits more microbes with lignolytic potential

To evaluate whether lignin-amended beads successfully recruited lignolytic organisms, we examined both oxidative enzymes assays as well as indicator species analysis of bead bacterial communities cross-referenced with a comparative literature search. We expected communities associated with lignin-amended beads to demonstrate greater potential to degrade model lignin compounds, and to contain a greater fraction of OTUs closely related to known lignin degraders compared to unamended beads.

**Figure 1:**
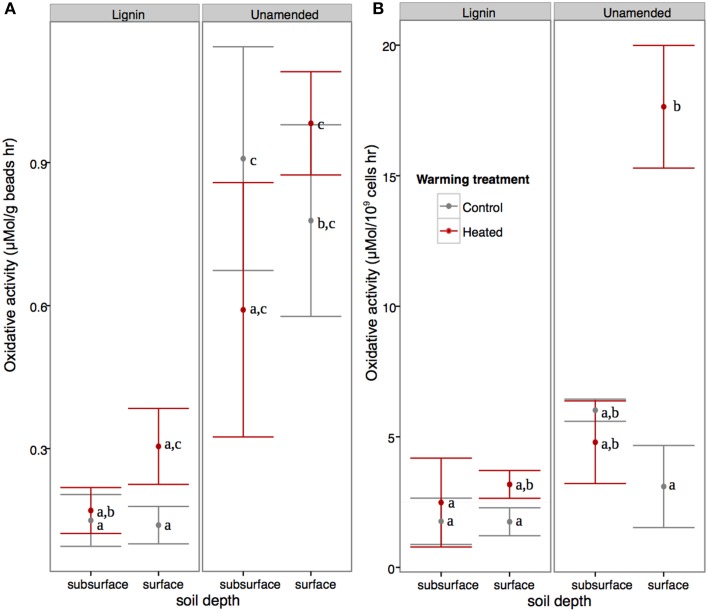
**Effect of warming and soil on field-moist bead (A) and cell-normalized total oxidative enzyme activity (B)**. Shared letters indicates soil depth*warming treatment*lignin amendment effects are not significantly different at *P* = 0.05 using Tukey's HSD.

In the surface beads, warmed communities showed 4.7x higher cell-normalized oxidative activity compared to controls, while in the subsurface beads there was no warming effect (Figure 1B; ANOVA warming^*^soil interaction *p* < 0.01, *F* = 8.69; three replicates used for surface lignin beads). Looking at the effect of lignin amendment, unamended beads showed approximately 4.5x greater bead weight normalized oxidative enzyme activity than lignin-amended beads (ANOVA, *p* < 0.001, *F* = 39.43, Figure 1A), and this was also true when accounting for differences in cell counts (ANOVA, *p* < 0.001, *F* = 17.65). DAPI-based cell counts of the enzyme slurry indicated that the number of cells per gram of beads was unaffected by the experimental factors (Three-Way full factorial ANOVA, *p* > 0.1 for all).

Sequencing of the 16S ribosomal RNA gene V4 region was used to identify the microbes associated with lignin-amended and unamended beads. Lignin-amended beads recruited many genera containing known lignin-degraders (Table 1). Overall, unamended beads had a greater fraction of reads assigned to genera with known lignin-degraders than lignin-amended beads did, but this was heavily skewed by *Burkholderia*, which accounted for almost 70% of the OTUs. Of the 27 genera we identified as containing known lignin-degrading taxa in a literature search, 15 had a significantly greater relative abundance in lignin than unamended beads, seven were unaffected by amendment, and five were enriched on unamended relative to lignin-amended beads (paired Wilcoxon, *p* < 0.05 for all). Those enriched for by lignin amendment included *Sphingomonas*, *Acinetobacter*, and *Agrobacterium*, respectively present at 6.4, 22, and 130x greater relative abundance in lignin than in unamended beads.

**Table 1:** **Relative abundance (reads per million reads) of genera with proposed lignin-degraders in lignin-amended and unamended bead samples**.

**Genus**	**Lignin-amended mean (SE)**	**Unamended bead mean (SE)**	***P*-value**	**References**
Acinetobacter	20700 (11900)	951 (468)	*p* < 0.05	Ghodake et al., 2009
Actinomyces	0 (0)	6.15 (4.59)	ns	Bugg et al., 2011a
Agrobacterium	78500 (35100)	595 (239)	*p* < 0.001	Deschamps et al., 1980
Amycolatopsis	1.92 (1.92)	18.8 (6.13)	*p* < 0.05	Bugg et al., 2011a
Arthrobacter	0 (0)	4.77 (3.51)	ns	Kerr et al., 1983
Bacillus	302 (200)	18.1 (7.78)	*p* < 0.05	Raj et al., 2007
Burkholderia	16000 (6470)	686000 (35700)	*p* < 0.001	Woo et al., 2014b
Caulobacter	1420 (527)	15.6 (8.27)	*p* < 0.01	Nierman et al., 2001
Comamonas	2520 (1840)	44.7 (29.8)	*p* < 0.01	Chen et al., 2012
Corynebacterium	1.97 (1.97)	16.3 (8.6)	ns	Deschamps et al., 1980
Cupriavidus	273 (231)	82.8 (75.1)	ns	Shi et al., 2013
Cytophaga	3700 (1400)	163 (103)	*p* < 0.01	Gonzalez et al., 1986
Klebsiella	22.0 (12.8)	2 (2)	ns	Deschamps et al., 1980; Woo et al., 2014a
Microbacterium	29.0 (8.97)	2 (2)	*p* < 0.05	Taylor et al., 2012
Nocardia	98.5 (39.7)	367 (120)	*p* < 0.01	Trojanowski et al., 1977
Ochrobactrum	9100 (5190)	877 (745)	*p* < 0.01	Taylor et al., 2012
Paenibacillus	6920 (2430)	107 (47.5)	*p* < 0.001	Chandra et al., 2008
Pandoraea	4440 (1550)	1200 (377)	ns	Chandra et al., 2007; Raj et al., 2007
Pseudomonas	26100 (11300)	4140 (1670)	*p* < 0.01	Odier et al., 1981; Bugg et al., 2011b
Rhizobium	6530 (2860)	1390 (1070)	*P* < 0.05	Gonzalez et al., 1986; Taylor et al., 2012
Rhodococcus	132000 (38400)	4140 (3190)	*p* < 0.001	Taylor et al., 2012
Serratia	1490 (799)	90 (33.1)	ns	Perestelo et al., 1990
Sphingobacterium	4960 (1460)	575 (318)	*p* < 0.001	Taylor et al., 2012
Sphingobium	1191 (441)	50.1 (21)	*p* < 0.01	Meux et al., 2012; Gall et al., 2014
Sphingomonas	20000 (4920)	3120 (761)	*p* < 0.05	Bugg et al., 2011a
Staphylococcus	0 (0)	16.1 (8.27)	*p* < 0.05	DeRito et al., 2005
Streptomyces	281 (143)	12500 (10600)	*p* < 0.05	Pasti et al., 1990

*SE is standard error of the mean. P-value results from a paired Wilcoxon-rank sum test corrected for multiple testing using Benjamini-Hochberg correction*.

### Warming was associated with increased diversity in bacterial communities

The bacterial communities in warmed plots were on average 32% richer (*p* < 0.05), 10% more even (*p* < 0.05), and had a phylogenetic diversity that was 28% greater (*p* < 0.01) than communities from control plots (Table 2). Examining communities associated with the lignin beads alone, warming increased just one metric of diversity, Faith's PD. There was no evidence for interactions between any of the experimental factors.

**Table 2:** **Alpha diversity of prokaryotic community based on rarefied community matrix**.

	**Diversity: Shannon's H [N_eff_ (Jost)]**	**Observed richness**	**Evenness: Shannon's evenness**	**Phylogenetic diversity: Faith's PD**
**Heated**	2110 (453)	5330 (1305)	0.849 (0.0223)	154 (9.12)
**Control**	862 (172)	4190 (926)	0.773 (0.0236)	120 (7.79)
Heat Treatment significance	^*^	^**^	^*^	^**^
**Lignin-amended**	1310 (359)	4250 (1190)	0.805 (0.0230)	118 (9.14)
**Unamended**	1670 (392)	5265 (1140)	0.816 (0.0267)	156 (7.29)
Lignin amendment significance	n.s.	^**^	n.s.	^***^
**Surface**	1280 (391)	4330 (1170)	0.791 (0.0270)	123 (8.31)
**Subsurface**	1690 (358)	5190 (1130)	0.831 (0.0215)	151 (9.33)
Soil depth significance	n.s.	^*^	n.s.	^**^

Means are reported ± standard error. P-values are indicated as p < 0.05 (^*^), p < 0.01(^**^), p < 0.001 (^***^), and not significant (n.s.).

### Bead amendment drives beta diversity among bacteria and fungi

**Figure 2:**
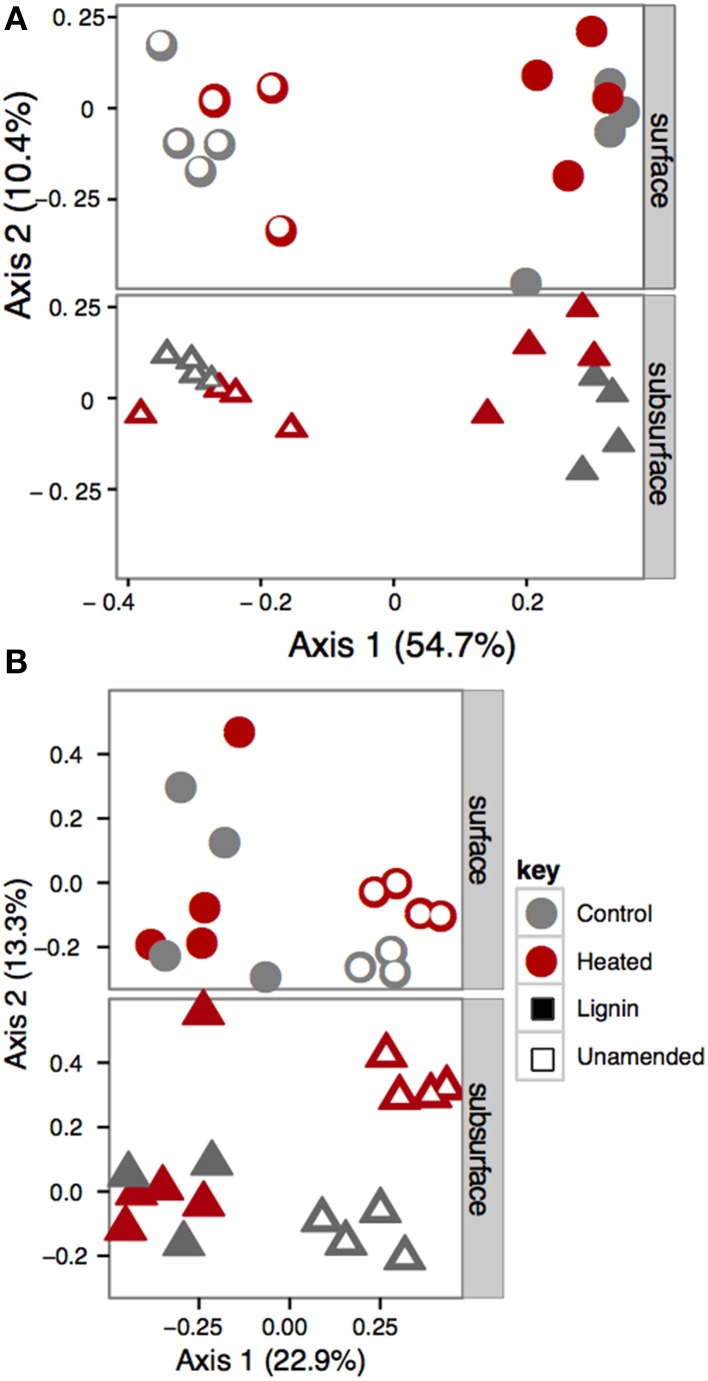
**Principal coordinates plot of (A) UniFrac distances of bacterial communities and (B) Hellinger distance of Fungal communities**. Lignin-amended and unamended beads support distinct communities, as demonstrated by the wide separation on the first axis.

Bead amendment was the primary force driving bacterial community structure, based on a permutational ANOVA of the UniFrac distances (*F* = 32.74; *p* = 0.001; *R*^2^ = 0.487; Figure 2A). Warming treatment (*F* = 3.93; *p* = 0.013; *R*^2^ = 0.059) played a secondary role in shaping overall bacterial beta diversity, with a greater fraction of the variation explained in the lignin-amended (*R*^2^ = 0.253) than the unamended beads (*R*^2^ = 0.141). We also analyzed factors driving relative abundance of each phylum individually (or subphyla, for the Proteobacteria) using step-down regression (Table S2). Consistent with whole community data, bead amendment was a primary driver of relative abundance of most phyla and subphyla considered, with Alphaproteobacteria at higher abundances on lignin-amended compared to unamended beads, and Acidobacteria, Actinobacteria, and Gammaproteobacteria at lower abundance on lignin-amended compared to unamended beads. Betaproteobacteria also tended to have reduced relative abundances in lignin-amended beads, but the fit for the model was poor (*R*^2^ adj = 0.19) compared to most of the other classes and phyla considered.

Fungal community structure was also driven by bead amendment (*F* = 8.75, *p* = 0.001, *R*^2^ = 0.209), warming treatment (*F* = 2.33; *p* = 0.008, *R*^2^ = 0.056), and soil effect (*F* = 2.10; *p* = 0.028; *R*^2^ = 0.050; Figure 2B), as determined by permutational ANOVA using Hellinger distance of the fungal TRFLP profiles (Blackwood et al., 2003). Warming (adonis, *F* = 2.85; *p* = 0.002; *R*^2^ = 0.153) and soil effect (*F* = 2.537; *p* = 0.002; *R*^2^ = 0.136) also played a significant role in structuring the fungal community in unamended beads when examined alone, but not lignin-amended beads.

### OTUs enriched on lignin-amended beads are phylogenetically clustered

Since soil depth was not a significant driver of bacterial beta diversity, we pooled samples by soil depth and plot to identify OTUs whose abundance was affected by lignin amendment and/or warming. Lignin-amended and unamended beads showed distinct communities at the level of the OTU, sharing just 9924 (17.5%) of the 56,835 total OTUs present. Examining the 9924 OTUs shared between lignin-amended and unamended beads and re-rarefying the community, we were able to identify 213 OTUs significantly enriched on lignin-amended beads compared to unamended beads, and 536 significantly enriched on unamended beads compared to lignin-amended beads (based on a Wilcoxon rank-sum test with corrections for multiple testing, Table S3). OTUs in the phylum Acidobacteria were exclusively enriched on unamended beads, and many more Actinobacteria OTUs were enriched on unamended than lignin-amended beads. By contrast, an approximately equal number of Betaproteobacteria OTUs were enriched on unamended as on lignin-amended beads; unamended beads were enriched in a number of members of the family Burkholderiaceae, while lignin-amended beads were enriched in OTUs of the family Comamonadaceae. This taxonomic trend was representative of the whole dataset; overall, Burkholderiaceae were 49.9x more abundant in the unamended than the lignin-amended beads, and Comamonadaceae were 9.2x more abundant in the lignin-amended compared to the unamended beads. The only orders that included members enriched only on the lignin-amended beads were the Cytophagales and Flavobacteriales (phylum Bacteroidetes, two and four OTUs, respectively), Bacilliales (three OTUs in the phylum Firmicutes), Planctomycetales (one OTU in the phylum Planctomycetes), Methylophilales (one OTU in the class Betaproteobacteria), Pseudomonadales (six OTUs in the class Gammaproteobacteria) (Table S3).

### OTUs enriched by warming on lignin beads are not phylogenetically clustered

**Figure 3:**
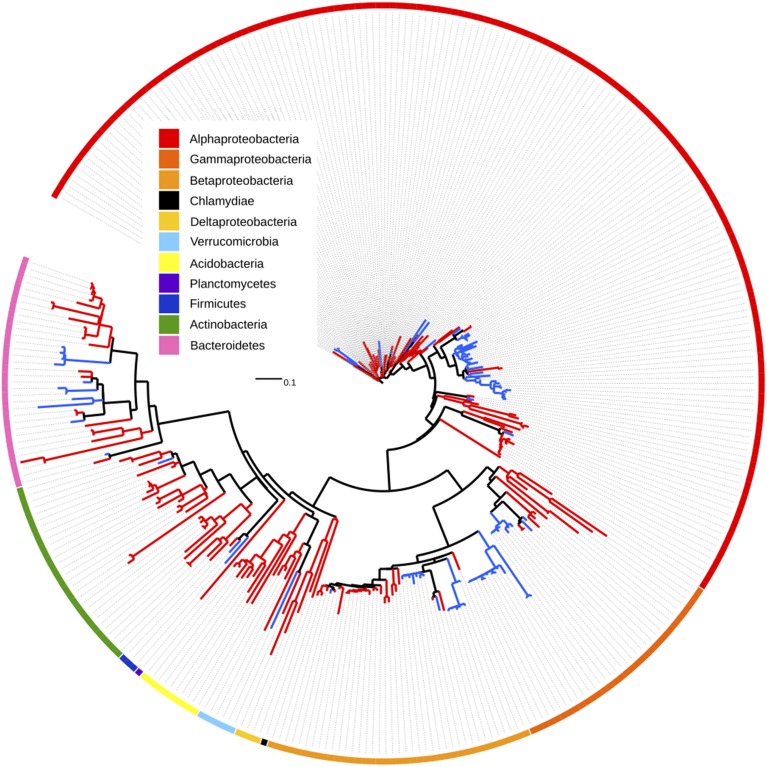
**Tree depicting OTUs which are significantly enriched in warmed (red branches) or control (blue branches) plots on lignin-amended BioSep beads**. OTUs also affected by warming treatment in the unamended beads were excluded from the tree. Each branch is a single OTU. Outer ring denotes phylum or class.

For the subset of 9924 OTUs shared by both bead types, we used indicator species analysis (IndVal, Cáceres and Legendre, 2009) with a Benjamini-Hochberg correction for multiple testing to identify those OTUs differentially affected by warming on unamended and lignin-amended beads. Overall, 109 OTUs were negatively affected by warming on lignin-amended beads, while 256 increased in relative abundance, and the majority of these showed a warming response that was unique to the lignin beads (Figure 3; Table S4). While many taxonomic groups showed mixed responses to warming, individual members of the phyla Acidobacteria and Actinobacteria, and families Bradyhizobiaceae, Sphingomonadaceae, and Comamonadaceae showed clear increases in abundance with warming on lignin-amended beads, while members of the Caulobacteraceae, Oxalobacteraceae and Moraxellaceae decreased in relative abundance.

## Discussion

In this study, we evaluated the hypothesis that two decades of experimental warming had selected for a microbial community better able to degrade chemically complex carbon. We based our expectation on observations that soil respiration at our experimental warming site has remained high despite considerable loss of soil organic matter (Melillo et al. in preparation) and of microbially available C (Bradford et al., 2008). To test this hypothesis, we used oxidative enzyme activity as a proxy for complex organic matter decomposition potential, and evaluated whether warming treatment affected the composition and diversity of the microbial community associated with lignin degradation.

The microbial communities subject to prolonged warming experienced reduced carbon availability (Bradford et al., 2008) and in this study showed increased total oxidative enzyme activity per billion cells, but the effect was only significant in the surface samples. Since there may be considerable overlap in pathways used to degrade lignin and soil organic matter (Fontaine et al., 2003; Carney et al., 2007), this result indicates the microbial community accessing complex carbon in the organic horizon was more strongly affected by warming treatment than the community accessing mineral-sorbed SOM. Microbial communities in the organic horizon are typically dominated by fungi (Baldrian et al., 2012), which are able to efficiently degrade lignin (Ahmad et al., 2010). However, fungal lipid biomass (Frey et al., 2008) and ITS region counts (DeAngelis et al., 2015) have been noted to be reduced by warming, so increased oxidative enzyme activity in surface soils with warming is unexpected. However, on a per gram basis, enzyme activity was unaffected by warming in surface bead samples, indicating that the smaller number of cells present in the heated samples may be more effective at degrading lignin-like compounds, a phenomenon that has been previously observed (Frey et al., 2013).

In our study, warming treatment preferentially affected microbes and oxidative activity in the surface compared to subsurface, where surface microbes are adapted to relatively “free” lignin compared to the mineral-stabilized SOM in the subsurface soils. However, since only half (9 of 16) of the subsurface bead bags were at the mineral-organic interface, it is difficult to directly assess this hypothesis. Factors such as a more stable temperature and moisture in the subsurface beads may have enabled microbes to invest more energy into producing extracellular enzymes, and less into maintenance than was possible for the surface beads. In addition to the showing greater enzymatic response to warming treatment, the surface beads were also home to a less phylogenetically-diverse bacterial community than the subsurface beads. This may in part be due to the increased moisture fluctuations faced by the surface beads, which may reduce diversity of the water-bound bacterial community (Lavelle, 1997).

Based on the fact that total oxidative enzyme activity was higher in unamended beads than lignin amended beads, it is unclear whether lignin amended Bio-Sep beads successfully recruited a lignolytic community. While it is possible that lignin was depleted on the lignin amended beads at the time of collection, we believe this is unlikely. In a similar experiment using these beads in tropical forest soils, DeAngelis et al. (2011) found that lignin amended beads had higher oxidative enzyme activity even after 30 weeks in the field, a time much longer than that used in the present study. Instead, higher oxidative enzyme activity in the unamended beads may be due to their better suitability for fungal colonization. DeAngelis et al. previously noted that fungi formed a smaller fraction of the eukaryotic community in lignin amended than unamended beads, and the small pores on the exterior of Bio-Sep beads have been reported to limit eukaryotic access (Williams et al., 2013). We observed that lignin amended beads are 44% denser than unamended beads, indicating that lignin amendment may have made it even more difficult for larger organisms to colonize the interior of the beads. Thus, the unexpected higher enzyme activity in unamended compared to lignin amended beads in our study may be indicative of the dominant lignin degraders being unable to access the substrate held within.

Lignin amended beads recruited a phylogenetically diverse collection of bacteria, including many in genera where known lignin degraders are found. These genera with significantly higher relative abundance in lignin amended than unamended beads included *Rhodococcus* and *Pseudomonas* but didn't include a number of the better-known lignin degrading genera such as *Streptomyces*, which are well-known for their biopolymer degrading capacity (Kampfer, 2012). *Rhodococcus jostii* RHA1 and *Pseudomonas putida* mt-2 are both able to degrade lignin without exogenous hydrogen peroxide and at a rate equivalent to some lignolytic fungi (Ahmad et al., 2010). We also found that *Burkholderia*, a cosmopolitan genus known for its diverse metabolism (Garrity et al., 2012), and *Nocardia*, one of the better-known lignin degraders (Bugg et al., 2011a), were present at significantly higher relative abundance in unamended than lignin amended beads. A mixture of OTUs in orders known to contain aromatic compound degraders, such as *Sphingobacteriales* (Taylor et al., 2012), and *Xanthomonadales* (Odier and Monties, 1978), and orders not previously noted to degrade lignin, such as *Saprospirales*, were found to be enriched in lignin compared to unamended beads. Likewise, warming treatment specifically enriched for OTUs in a combination of orders with and without known lignin degraders. For instance, eight members of Acidobacteria were enriched by warming on lignin beads, although to our knowledge direct evidence for their ability to degrade lignin has not yet been reported. It is also possible that their enrichment is due to ability to degrade lignin by-products produced by other lignin-degrading organisms. Dedysh and colleagues isolated Acidobacteria from phenolic-rich peats and mosses using humic acid (Dedysh et al., 2006; Pankratov and Dedysh, 2010), indicating these organisms may have lignolytic potential. However, it is probable that our lignin beads recruited organisms feeding on lignin decomposition products, rather than lignin, *per se*. A recent paper also noted the possibility that organisms in lignin-rich environments may gain the ability to degrade complex carbon sources through rapid horizontal gene transfer (Strachan et al., 2014).

In addition to specifically selecting for potential lignin degraders, warming may enhance the ability of the microbial community to degrade chemically complex carbon by increasing overall community diversity. Increased community diversity is associated with the acceleration of multiple ecosystem processes, including decomposition and respiration (Strickland et al., 2009; Cadotte et al., 2012; Pold and DeAngelis, 2013). However, across warming studies, the response of microbial community diversity and composition to experimental warming treatment has been variable (Pold and DeAngelis, 2013). At our study site, the community profile based on FAMEs had shifted after 12 years of warming in the mineral horizon (Frey et al., 2008). When first studied using sequencing after 20 years of warming, the bacterial community was noted to be more diverse overall and to have shifted in composition in the organic horizon (DeAngelis et al., 2015). Thus, the increase in diversity and altered community structure observed here for the bacterial and fungal communities was in line with previous results at our site. It remains unclear whether the diversity or identity of lignin-associated bacteria seen here may be driving observed changes in carbon cycling in our site.

Ultimately, the ability of the microbial community to efficiently convert soil carbon to biomass is the key first step in the release of stored soil carbon (Allison et al., 2010). Chemically complex compounds such as lignin are generally utilized by microbes at lower efficiencies than simpler ones (Manzoni et al., 2012), and microbes can differ in the efficiency with which they utilize litter carbon sources. Previous observations that the warmed plot microbial community at our site is adapted to more efficient use of phenolic compounds than that of the control plots (Frey et al., 2013) lead to the hypothesis that changes in microbial communities captured on our lignin beads may be contributing to the altered fate of carbon in soil. Though the lignin in our beads was not under the same physical and chemical constraints for degradation that soil organic matter may be, the potential overlap in degradation pathways for complex soil organic matter and lignin indicates that at least some of the organisms preferentially enriched for on lignin-amended beads are likely to also degrade soil organic matter. Yet we also recognize that the presence of an organism in a lignin-amended bead may be indicative of tolerance to oxidative stress as well as ability to degrade the lignin present (DeAngelis et al., 2011). Because of this, we refrain from drawing conclusions in the comparison of the lignin-amended compared to the unamended beads. However, the diversity of observed bacterial genera that were recruited to the lignin-amended beads, and the consistency of the taxonomy of many of these organisms with known lignin degraders, indicates that we have captured a functionally important subset of the community.

## Conclusions

We found evidence for a change in the structure of the bacterial community adapted to degradation of a major litter component, lignin, as we have seen for the “whole-soil” bacterial community after two decades of warming at this site (DeAngelis et al., 2015). While we cannot conclude that the continued loss of soil carbon at our study site is due to the changes in the microbial community observed here, our research is consistent with previous research (Frey et al., 2013) in indicating that those microbes potentially capable of degrading complex carbon substrates are at least responsive to the direct or indirect effects of two decades of elevated temperature. Ultimately, the fate of soil organic matter in a warming world will depend upon a complex interplay between the efficiency with which the microbial community converts litter to biomass, and the ease with which this biomass is recycled into new biomass or physically stabilized by the soil matrix. Future approaches to partition enzymes to domains, as well as an assessment of the temperature sensitivity of oxidative enzyme activity, will help elucidate mechanisms driving observed increases in rates of soil organic matter loss at our site.

### Conflict of interest statement

The authors declare that the research was conducted in the absence of any commercial or financial relationships that could be construed as a potential conflict of interest.
