# Supplementary material for: Two decades of warming increases diversity of a potentially lignolytic bacterial community
Source: Front Microbiol. 2015 May 20;6:480. doi: 10.3389/fmicb.2015.00480 (PMC4438230; doi:10.3389/fmicb.2015.00480)
Supplement: Supplementary file 1 [file Presentation1.PDF]

Supplementary information captions:

I. Tables

Table S1: Sequencing statistics for each step of the data processing pipeline

Table S2: Best-fit models for dominant taxa in BioSep beads using step-down regression.

Table S3: Summary of OTUs significantly enriched in lignin-amended or unamended BioSep beads

Table S4: indicator OTUs of warming treatment.

Table S1. Sequencing statistics for each step of the data processing pipeline:

Run	Bead OTUs	Bead Observations (count)	Notes
Total reads	n.a.	20,548,038	Total reads after sequencing
Post-assembly	n.a.	9,829,999	Using FLASH aligner and after size selection
Quality filtering	n.a.	9,480,366	After split_libraries_fastq.py
Open, 99%, raw	152,560	8,937,796	Open referenced OTU calling (99%), no singletons
Open, 99%	130,108	8,646,121	No singletons, chimeras excluded
Open, 99%, no2s	64,351	8,514,607	No singletons or doubletons, chimeras excluded
Open, 99%, no2s, rarefied	56,835	2,640,000	No singletons or doubletons, chimeras excluded, samples rarefied to observations in smallest sample
Open, 99%, >0.1%	110	5,286,199	Dominants (>0.1% relative abundance (>8514))
Open, 99%, >0.1%, rarefied	110	1,281,664	Dominants (>0.1% relative abundance (>8514)), rarefied

Table S2. Best-fit models for dominant taxa in BioSep beads using step-down regression, where variables are abbreviated as warming treatment "H," lignin-amendment "A," and soil effect "W," with interaction terms shown as both variables concatenated. Reference levels (0) are control (warming treatment), lignin (bead amendment), and subsurface (soil depth).

Taxon	Formula	Adjusted R^2	P value
Acidobacteria	Y = -0.011H + 0.016W + 0.054A – 0.022HA + 0.005	0.640	p<0.0001
Actinobacteria	Y = -0.029H + 0.078A + 0.169HA + 0.117	0.421	p<0.001
Alphaproteobacteria	Y = -0.332A + 0.455	0.678	p<0.0001
Betaproteobacteria	Y = 0.118A + 0.240	0.195	p<0.01
Gammaproteobacteria	Y = -0.033W + 0.051A + 0.077	0.335	P<0.01
Bacteroidetes	Y = -0.073A + 0.105	0.542	P<0.0001
Verrucomicrobia	Y = 0.013A + 0.010W + 0.002H + 0.013AW – 0.0156HA + 0.004	0.376	P<0.01
Planctomycetes	Y = 0.003A + 0.010W – 0.010HA + 0.003	0.200	P<0.05

Table S3: Summary of OTUs significantly enriched in lignin-amended or unamended BioSep beads based on a paired Wilcoxin rank-sum test with Benjamini-Hochberg correction for multiple testing ($p<0.05$). Rows are total number of significantly enriched OTUs at given taxonomic level. Phylum-level sums are bolded, while class-level sums are italicized

			Higher in lignin	Higher in unamended
p__Acidobacteria			**0**	**101**
	c__Acidobacteria	o__Acidobacteriales	0	86
	c_DA052	o_Ellin6513	0	7
	c__Solibacteres	o__Solibacteres	0	8
p__Actinobacteria			**9**	**121**
	c__Acidimicrobiia	o__Acidimicrobiales	0	20
	c__Actinobacteria	o__Actinomycetales	9	75
	c__Thermoleophilia		*0*	*26*
		o_Gaiellales	0	2
		o__Solirubrobacterales	0	24
p__Armatimonadetes			**0**	**8**
	c__[Fimbriimonadia]	o__[Fimbriimonadales]	0	4
	c_Armatimonadia		*0*	*4*
		o__Armatimonadales	0	1
		o__FW68	0	3
p__Bacteroidetes			**37**	**29**
	c__[Saprospirae]	o__[Saprospirales]	10	6
	c__Cytophagia	o__Cytophagales	2	0
	c__Flavobacteriia	o__Flavobacteriales	4	0
	c__Sphingobacteriia	o__Sphingobacteriales	21	23
p__Firmicutes	c__Bacilli	o__Bacillales	**3**	**0**
p__Planctomycetes			**1**	**2**
	c__Planctomycetia		*1*	*2*
		o__Planctomycetales	1	0
		o__Gemmatales	0	2
	c__vadinHA49	o__DH61	0	1
p__Proteobacteria			**162**	**246**
	c__Alphaproteobacteria		*77*	*110*
		o__	0	5
		o__Caulobacterales	11	14
		o__Ellin329	0	13
		o__Rhizobiales	58	32
		o__Rhodospirillales	3	45
		o__Sphingomonadales	5	1
	c__Betaproteobacteria		*71*	*87*
		o__Burkholderiales	70	87
		o__Methylophilales	1	0
	c__Deltaproteobacteria		*1*	*5*
		o__Myxococcales	1	4
		o__Syntrophobacterales	0	1
	c__Gammaproteobacteria		*13*	*44*
		o__Legionellales	2	1
		o__Pseudomonadales	6	0
		o__Xanthomonadales	5	43
p__Verrucomicrobia			**1**	**29**
	c__[Methylacidiphilae]	o__Methylacidiphilales	0	5
	c__[Pedosphaerae]	o__[Pedosphaerales]	0	17
	c__[Spartobacteria]	o__[Chthoniobacterales]	0	5
	c__Opitutae	o__Opitutales	1	2
p__WPS-2	c__	o__	**0**	**4**
TOTAL			213	536

Table S4: indicator OTUs of warming treatment. Numbers denote the number of OTUs present at significantly higher abundance in beads incubated in warmed (W) or control (C) plots. Numbers in brackets are the number of OTUs for which showed a similar significant warming trend in both bead types, while those without are unique in their response.

				lignin		unamended	
phylum	class	order	family	Control	Warmed	Control	Warmed
p__Acidobacteria	c__Acidobacteriia	o__Acidobacteriales	f__Acidobacteriaceae	-	7	5	18
			f__Koribacteraceae	-	1	-	6
	c__DA052	o__Ellin6513	f__	-	1	-	4
	c__Solibacteres	o__Solibacterales	f__	-	1	-	4
p__Actinobacteria	c__Acidimicrobiia	o__Acidimicrobiales	f__	-	4	2	3
	c__Actinobacteria	o__Actinomycetales	f__	-	6 (2)	1	0 (2)
			f__Actinospicaceae	-	-	2	2
			f__Actinosynnemataceae	-	-	-	1
			f__Frankiaceae	-	1	-	2
			f__Gordoniaceae	-	2	-	1
			f__Microbacteriaceae	1	-	3	-
			f__Mycobacteriaceae	-	2	1	-
			f__Nocardiaceae	-	1	-	-
			f__Nocardioidaceae	-	4	1	-
			f__Pseudonocardiaceae	-	1	-	2
			f__Streptomycetaceae	-	-	1	-
			f__Thermomonosporaceae	-	-	-	1
	c__Thermoleophilia	o__Gaiellales	f__Gaiellaceae	-	1	-	-
		o__Solirubrobacterales	f__	-	1	2	2
			f__Conexibacteraceae	-	4	-	4
			f__Patulibacteraceae	-	-	1	-
p__Armatimonadetes	c__[Fimbriimonadia]	o__[Fimbriimonadales]	f__[Fimbriimonadaceae]	-	-	-	4
	c__Armatimonadia	o__Armatimonadales	f__Armatimonadaceae	-	-	-	1
		o__FW68	f__	-	-	-	2
	c__Chthonomonadetes	o__Chthonomonadales	f__Chthonomonadaceae	-	-	-	1
p__Bacteroidetes	c__[Saprospirae]	o__[Saprospirales]	f__Chitinophagaceae	4	12 (2)	1(1)	0(1)
	c__Cytophagia	o__Cytophagales	f__Cytophagaceae	-	5	-	-
	c__Flavobacteriia	o__Flavobacteriales	f__[Weeksellaceae]	1	1	1	1
	c__Sphingobacteriia	o__Sphingobacteriales	f__	-	1	-	2
			f__Sphingobacteriaceae	6	4	8	8
p__Chlamydiae	c__Chlamydiia	o__Chlamydiales	f__Rhabdochlamydiaceae	-	1	1	-
p__Elusimicrobia	c__Elusimicrobia	o__FAC88	f__	-	-	-	2
p__OD1	c__SM2F11	o__	f__	-	-	-	1
	c__ZB2	o__	f__	-	-	1	-
p__Firmicutes	c__Bacilli	o__Bacillales	f__Paenibacillaceae	2	1	-	-
p__Planctomycetes	c__Planctomycetia	o__Gemmatales	f__Gemmataceae	-	1	-	6
			f__Isosphaeraceae	-	2	-	1
	c__Phycisphaerae	o__WD2101	f__	-	-	1	-
p__Proteobacteria	c__Alphaproteobacteria			-	1	-	1
		o__Caulobacterales	f__Caulobacteraceae	21	2	-	5
		o__Ellin329	f__	-	1	1	4
		o__Rhizobiales	f__	3	8	-	-
			f__Aurantimonadaceae	11	-	-	-
			f__Beijerinckiaceae	1	1	-	-
			f__Bradyrhizobiaceae	1	72 (4)	-	13 (4)
			f__Brucellaceae	2	-	-	-
			f__Hyphomicrobiaceae	-	13 (1)	-	5(1)
			f__Methylobacteriaceae	-	4	-	-
			f__Methylocystaceae	-	2	-	10
			f__Phyllobacteriaceae	6	-	-	-
			f__Rhizobiaceae	7	4	-	-
			f__Xanthobacteraceae	-	5 (1)	-	0 (1)
		o__Rhodobacterales	f__Rhodobacteraceae	1	-	-	-

		o__Rhodospirillales	f__Acetobacteraceae	1	1	-	7
			f__Rhodospirillaceae	-	1	1	3
		o__Sphingomonadales	f__	-	1	-	-
			f__Sphingomonadaceae	1	11 (1)	-	0 (1)
			f__	-	1	-	-
			f__Alcaligenaceae	6	-	-	-
	c__Betaproteobacteria	o__Burkholderiales	f__Burkholderiaceae	-	5	2	8
			f__Comamonadaceae	1	19	3	-
			f__Oxalobacteraceae	9	1	2	1
		o__Bdellovibrionales	f__Bdellovibrionaceae	-	1	-	-
			f__	-	2 (1)	-	6 (1)
	c__Deltaproteobacteria	o__Myxococcales	f__0319-6G20	-	-	-	2
			f__Haliangiaceae	-	1	-	-
			f__Myxococcaceae	-	2	-	2
		o__Syntrophobacterales	f__Syntrophobacteraceae	-	-	-	1
		o_	f_	-	-	-	1
		o__Enterobacteriales	f__Enterobacteriaceae	2	-	-	-
			f__	-	1	1	-
	c__Gammaproteobacteria	o__Legionellales	f__Coxiellaceae	-	4	4	2
			f__Legionellaceae	-	-	-	2
		o__Pseudomonadales	f__Moraxellaceae	10	-	-	2
			f__Pseudomonadaceae	-	-	3	-
		o__Xanthomonadales	f__Sinobacteraceae	-	3	-	7
	c__[Methylacidiphilae]		f__Xanthomonadaceae	11	5 (1)	8	4(1)
			f__	-	-	-	3
	c__[Pedosphaerae]	o__[Pedosphaerales]	f__[Pedosphaeraceae]	-	-	-	3
			f__auto67_4W	-	-	-	7
p__Verrucomicrobia	c__[Spartobacteria]	o__Methylacidiphilales	f__	-	1	1	5
	c__Opitutae	o__[Chthoniobacterales]	f__[Chthoniobacteraceae]	-	3	-	2
	c__Verrucomicrobiae	o__Opitutales	f__Opitutaceae	-	1	-	1
		o__Verrucomicrobiales	f__Verrucomicrobiaceae	1	1	-	-
p__WPS-2	c__	o__	f__	-	-	-	3
Total				109	117	66	163
